# Hazard Analysis of Critical Control Points Assessment as a Tool to Respond to Emerging Infectious Disease Outbreaks

**DOI:** 10.1371/journal.pone.0072279

**Published:** 2013-08-14

**Authors:** Kelly L. Edmunds, Paul R. Hunter, Roger Few, Diana J. Bell

**Affiliations:** 1 Centre for Ecology, Evolution and Conservation, School of Biological Sciences, University of East Anglia, Norwich, Norfolk, United Kingdom; 2 Norwich Medical School, University of East Anglia, Norwich, Norfolk, United Kingdom; 3 School of International Development, University of East Anglia, Norwich, Norfolk, United Kingdom; Alberta Provincial Laboratory for Public Health/ University of Alberta, Canada

## Abstract

Highly pathogenic avian influenza virus (HPAI) strain H5N1 has had direct and indirect economic impacts arising from direct mortality and control programmes in over 50 countries reporting poultry outbreaks. HPAI H5N1 is now reported as the most widespread and expensive zoonotic disease recorded and continues to pose a global health threat. The aim of this research was to assess the potential of utilising Hazard Analysis of Critical Control Points (HACCP) assessments in providing a framework for a rapid response to emerging infectious disease outbreaks. This novel approach applies a scientific process, widely used in food production systems, to assess risks related to a specific emerging health threat within a known zoonotic disease hotspot. We conducted a HACCP assessment for HPAI viruses within Vietnam’s domestic poultry trade and relate our findings to the existing literature. Our HACCP assessment identified poultry flock isolation, transportation, slaughter, preparation and consumption as critical control points for Vietnam’s domestic poultry trade. Introduction of the preventative measures highlighted through this HACCP evaluation would reduce the risks posed by HPAI viruses and pressure on the national economy. We conclude that this HACCP assessment provides compelling evidence for the future potential that HACCP analyses could play in initiating a rapid response to emerging infectious diseases.

## Introduction

Since 1980, on average one new emerging infectious disease (EID) has appeared in humans every eight months [1] with the emergence of these pathogenic infectious diseases representing a substantial global threat to human health [2,3]. Research has found that of all EIDs, 60.3% are zoonoses originating in wildlife and these represent the most significant global health threat [4–6]. Examples of key recent EID outbreaks include Ebola haemorrhagic fever [5,7], SARS coronavirus [8], highly pathogenic avian influenza (HPAI) and most recently, avian influenza A H7N9 [9].

Since late 2003, highly pathogenic avian influenza virus (HPAI) strain H5N1 has been responsible for the deaths of millions of animals, primarily poultry taxa but also a range of other avian and mammalian species [9,10]. HPAI H5N1 has been reported in poultry from over 50 countries with 375 human deaths among 630 confirmed cases (59. 5 % confirmed case fatality risk) recorded in 12 of these countries as of 30 June 2013 [9,11]. The countries of Southeast Asia have been hardest hit by HPAI H5N1 with 2681 reported outbreaks in domestic poultry in Vietnam alone by 30 June 2013. In the first five months of 2013, 15 human fatalities have occurred in the 20 cases of HPAI H5N1 so far been reported in Cambodia, China and Egypt [11].

Approximately 80% of the Vietnamese population live in rural areas and almost 80% of these rural households participate in small-scale (backyard) poultry production [12]. The Red River and Mekong River deltas are major poultry producing areas from which poultry and their products (e.g. eggs, faeces, feathers) may be transported directly to the point of sale by the breeder or pass through a number of middle-men in the trade chain.

HPAI H5N1 spread rapidly from Southeast Asia into Europe and Africa. The main mechanism for HPAI spread is the movement of poultry and their products [13,14] however the modes of poultry-to-human transmission of this virus remain poorly understood [15]. Live poultry markets are acknowledged as a reservoir for the virus within the Southeast Asia region [14,16].

Here we take a technique, Hazard Analysis of Critical Control Points (HACCP) analysis [17] and apply it to HPAI viruses within Vietnam’s poultry trade system to explore the role that this approach may have in catalysing efforts to tackle emerging infectious disease outbreaks. We identify the key stages within the poultry trade chain which pose risks for the transmission of HPAI viruses in human and poultry populations. We then discuss the potential use of HACCP assessments as a rapid response tool during the early stages of emerging infectious disease outbreaks, as a precursor to more time-consuming quantitative data collection and biomedical testing.

## Methods

**Figure 1:**
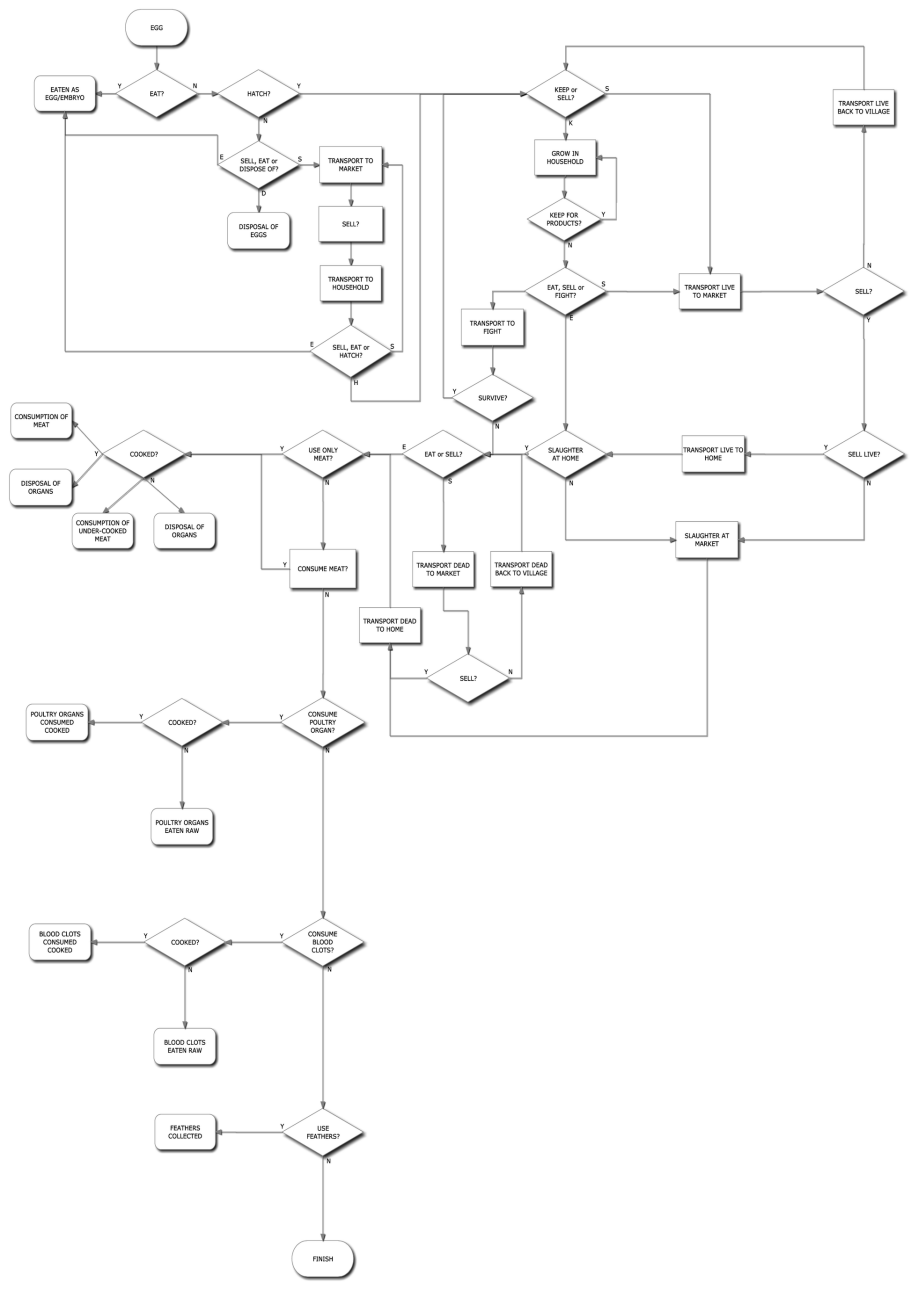
Flow chart used during the Hazard Analysis of Critical Control Points assessment conducted for the domestic poultry trade within Vietnam.

The HACCP assessment of Vietnam’s domestic poultry trade followed the first three HACCP principles (described in Table 1) to address our aims [17]. The initial flow chart created during the first stage of the HACCP assessment (see Figure 1) was developed based on our long-term research of Vietnam’s poultry trade. The flow chart begins with a poultry egg and tracks all the possible routes that this egg could take through Vietnam’s domestic poultry trade. This flow chart was then presented to a range of experts for critical analysis; including public health professionals, epidemiologists and wildlife disease biologists. A hazard was considered to be a process within Vietnam’s poultry trade providing an opportunity, at an unacceptable level of risk, for the transmission of HPAI either from poultry-to-human or poultry-to-poultry. Based on the frequency with which these hazards occur, they were then grouped into categories of posing a high or low-risk to poultry and/or humans.

**Table 1:** The first three principles of a Hazard Analysis of Critical Control Points **assessment**.

	**Aims**	**Actions**
**Principle 1**	Outline key ‘risk’ stages in system under investigation.	Conduct hazard analysis. Create flow chart of stages involved within the system in question and validate the flow chart through liaison with experts.
**Principle 2**	Identify Critical Control Points (CCPs) within the system	Critical review of the system to highlight stages which can adopt mitigation strategies for hazards known to occur frequently.
**Principle 3**	Develop CCPs and control recommendations for the recognised hazards	Ascertain critical limits for the CCPs identified and use these to generate recommendations for the improvement of the overall system.

Following the validation of the flow chart, we referred to the team of experts again to determine appropriate Critical Control Points (CCPs). A CCP is a point in poultry trade which provides an opportunity to control, prevent or eliminate the risks for HPAI transmission. Each of these first two principles required cross-referencing outputs with existing literature on HPAI virus epidemiology within Vietnam’s poultry trade. We also referred to recent reviews of the scientific literature to identify any risks which the HACCP analysis failed to identify.

Critical limits were then set for each of the CCPs identified. These critical limits are thresholds used as preventative measures to control the hazards within the system. Setting the critical limits required prior research of both Vietnam’s domestic poultry trade and consumer behaviour.

## Results

### Hazard Analysis

The stages of the poultry trade chain identified as presenting increased opportunities for HPAI transmission in the HACCP were grouped into four categories, namely: 1. contact within poultry flocks, 2. poultry transportation and sale, 3. poultry purchase and slaughter, and 4. poultry preparation and consumption.

1 Contact within poultry flocks occurs at multiple stages within the trade. These potential viral ‘mixing pots’ exist when i) established flocks mix with newly recruited birds purchased by the owner; ii) flocks mix at a market; iii) birds mix at communal HPAI H5N1 vaccinations centres and iv) fighting cock contests bring birds together in one contact arena. Each of these scenarios present high-risk opportunities for poultry to poultry transmission whereas scenarios i), iii) and iv) also present high-risk opportunities for poultry-to-human transmission.
2 Poultry may experience multiple transportation events across a large spatial scale throughout their lifetime. At all stages of the poultry trade, the transportation and sale of eggs, chicks, adult birds or poultry products, creates opportunities for human-mediated transmission of HPAI viruses. Due to the contact opportunities and volume of birds moved across various spatial scales, the transportation and sale of poultry is considered a high-risk activity for HPAI transmission from both poultry-to-poultry as well as poultry-to-humans.
3 The purchase and slaughtering of poultry from wet markets primarily occurs in one of two ways; i) purchase from wet markets can involve the consumer buying a live bird which they then take home to slaughter themselves or ii) they can request the poultry seller to slaughter and prepare a chosen bird which the consumer then takes home as joints of raw meat. Both of these modes of purchase are closely linked to the fourth risk category, described further below. Purchase of poultry via mode i) is an HPAI transmission risk to poultry if the consumer has other household poultry and a risk to the consumer themselves when they come to slaughter and prepare the bird at home. The purchase of poultry via mode ii) is an HPAI transmission risk for the poultry seller as they slaughter and prepare the bird at the market and the handling of raw meat is a transmission risk for the consumer.
4 The preparation of poultry for consumption introduces poultry–to-human HPAI transmission risks in the later stages of the trade chain, primarily through the slaughtering process. In the absence of appropriate hygiene practices, poultry slaughtering and carcass preparation put the slaughterer at substantial risk of exposure to HPAI viruses due to the contact with raw poultry and blood.

Poultry consumption (of meat, eggs, organs and blood from both chickens and ducks) is a high-risk activity for HPAI transmission from poultry-to-humans if the infection is maintained in the raw or under-cooked tissue. Contrastingly, the consumption of well-cooked poultry and poultry products pose low-risks for viral transmission.

### Critical Control Points and Critical Limits

CCPs were defined for each of the four risk stages identified during the HACCP assessment of Vietnam’s poultry trade. Each CCP is a point in the poultry trade which provides HPAI viruses with an opportunity for transmission between host animals. For each CCP, critical limits have been proposed to limit virus transmission risks from poultry-to-poultry and from poultry-to-humans (Table 2).

**Table 2:** Risk stages, critical control points and proposed critical limits identified through HACCP assessments for highly pathogenic avian influenza (HPAI) H5N1 transmission via Vietnam’s poultry trade.

**Risk Stage**	**Critical Control Point**	**Critical Limits**
***1.Contactwithinpoultryflocks***		
i) Newly recruited birds introduced into established flocks	Introduction of ‘foreign’ birds to an established flock^p^	Flock vaccination
ii) Awaiting sale at market	Arrival at market^p^	Flock isolation, quarantine newly-purchased birds
iii) Awaiting transport back to household	Arrival/preparation for departure^p^	Flock isolation, quarantine
iv) Communal poultry vaccination centres	Throughout vaccination^p,h^	Flock isolation, quarantine
v) Fighting cock contests	Throughout contest^p,h^	Isolation of birds, quarantine
***2.Poultrytransportation&sale***		
Transportation of live birds	Transfer from household^p^	Flock isolation throughout
Transportation of fighting cocks post-bout	Transfer post-fight^p,h^	Isolation
***3.Poultrypurchase&slaughter***		
Slaughter of birds	Carcass disposal^h^, poultry slaughter, collection of blood^h^	Use protective equipment, follow protocols, avoid direct contact
***4.Poultrypreparation&consumption***		
Consumption of under-cooked meat and eggs	Cooking^h^	Cook thoroughly
Contact with raw meat	Poultry preparation^h^	Use protective equipment, follow hygiene protocols, avoid direct contact

^p^ denotes the Critical Control Point (CCP) is intended to reduce the opportunity for poultry-to-poultry HPAI transmission, ^h^ denotes the CCP is intended to reduce the opportunity for poultry-to-human HPAI transmission.

The CCPs for limiting transmission through contact within poultry flocks involve the same approach as those for the transportation and sale of poultry; a combination of flock isolation, whereby established poultry flocks are prevented from mixing with other birds; quarantining newly purchased birds, where the newly purchased birds are held in isolation from other birds for a minimum of seven days; and household vaccination programmes (Table 2).

CCPs for the transportation and sale of the poultry begin once they depart from their household of origin. The suggested critical limit for this transmission risk is a total ban on inter-flock mixing of birds throughout poultry transportation and sale.

Preparing poultry for human consumption is the first stage of the trade chain when non-farmers are introduced to a high-risk opportunity to contract HPAI viruses. Two key CCPs concern poultry slaughtering and carcass preparation; this refers to the slaughter of poultry both at home and in the wet markets.

The associated risks can be reduced through the correct use of protective equipment such as face masks, gloves and sterile utensils to prevent contact with raw and bloody poultry. Further intervention should include the provision of additional education to the population through a range of health promotion mechanisms (including social media) as to how to handle potentially infectious meat to hygiene standards imposed as a feature of standard food preparation HACCPs in the retail food industry.

Poultry consumption is not a substantial risk for poultry-to-human HPAI transmission provided poultry products are well-cooked, thus the cooking stage is the CCP for poultry consumption with a critical limit of cooking temperature and duration. Consuming raw blood pudding poses some of the highest risks for poultry-to-human transmission of HPAI viruses and controlling this risk is only possible through thorough cooking practices or abstinence.

## Discussion

Our HACCP assessment has identified poultry flock isolation as well as the transportation, slaughter, preparation and consumption of poultry as critical control points for HPAI H5N1 transmission in Vietnam’s domestic poultry trade. Critical limits at each of these control points are recommended to control the risks of HPAI transmission from poultry-to-poultry and from poultry-to-humans.

The scope of Vietnam’s poultry trade is far-reaching both geographically and across social classes. Rural Vietnamese households typically keep a few backyard poultry and are likely to consume these birds or birds from neighbouring flocks. In urban Vietnamese households, it is less common for poultry to be kept within the household and birds are typically purchased at local markets [18]. Typically, the live poultry trade is dominated by birds sold with no animal health certification and which have been produced under questionable hygiene conditions.

Poultry provides an important source of income as well as a low-cost protein source for many rural Vietnamese households [18]. The HPAI H5N1 epidemic has been both a public health and an economic problem for many Vietnamese people particularly the rural poor when pre-emptive culling has been one of key interventions against H5N1.

If the management of infectious zoonotic diseases is to be successfully implemented, controlling the transmission chain from infected to uninfected animals is essential [13]. Pathogen spread between rural poultry flocks may occur when these mix or birds are moved within live markets, communal vaccination centres or during transportation and fighting cock contests [19].

Incubation periods differ between chickens and ducks with reports of deaths occurring within one to five days for chickens and up to seven days for ducks [20]. As a result, at all stages, flocks which have mixed with other poultry flocks should undergo a week-long quarantine period, after which, asymptomatic birds can be released. It has been noted that transmission of HPAI H5N1 between poultry appears to have shifted from the faecal/oral route towards the respiratory route [13] which underlines the risks of mixing poultry flocks.

Poultry and its products are often transported in considerable numbers across large spatial scales. During transportation, HPAI material may be shed by infected individuals and lead to other poultry coming into contact with viral material. Consultation of published literature has shown that exposure to an environment contaminated with viral material (for example via soil, fomites, feathers, water etc.) can also pose a risk for viral transmission [21–23]. The mixing and movement of poultry through ‘wet’ markets (those selling live animals) is known to play an important role in the transmission and spread of HPAI viruses [16,24]. It has been reported that exposure to live poultry at wet markets increases human/ HPAI H5N1 exposure four-fold [25]. Within some wet bird markets such as those in Hong Kong, current successful control programmes for HPAI viruses incorporate ‘rest days’ whereby the markets are closed and poultry stalls cleaned [16]. Implementation of such a practice is recommended in Vietnam’s cities.

The H5N1 virus can survive in poultry carcasses kept at room temperature for several days or longer at cooler temperatures [26]. Human infection with the H5N1 virus is associated with recent exposure to live poultry [25], direct contact with dead poultry [27] and the preparation or cooking of sick or dead poultry [28–30]. As a result, poultry market workers and poultry slaughterers are at particular risk of human HPAI H5N1 infection [24].

Within Vietnamese households it is typical to consume the meat, eggs and organs of both chickens and ducks. The consumption of chicken and chicken products varies from that of ducks with regard to the parts consumed [31]. Uncooked duck blood is commonly consumed for special occasions, a practice that has been implicated in poultry to human HPAI transmission [21,28].

Exposure to hazards iii) and iv) will depend on the vaccination system employed and the suitability of the birds for cock-fighting. In some communes the Department of Animal Health (DAH) organises door-to-door vaccinations by local veterinarians. In more remote villages, the DAH organises communal vaccination days where households from several villages bring their poultry to one centralised location for vaccination. This latter vaccination system encourages the mixing of poultry flocks from different localities, promoting contact within poultry flocks, and given the time-lag before the HPAI H5N1 vaccine becomes effective, presents a high risk for the transmission of HPAI viruses. Door-to-door vaccinations ensure a lower risk of inter-flock viral transmission and are recommended. Should this approach prove impractical, the isolation of flocks whilst at the communal vaccination centre would limit the chances of inter-flock viral transmission. It is also noted that vaccination programmes are currently lacking any system of coordinated monitoring [13] which would reduce virus spread.

Fighting cock contests may play a role in the transmission of HPAI viruses to humans [14,28]. Fighting cocks are valuable possessions and owners may transport birds large distances to participate in bouts and even lick the wounds sustained by their fighting cocks [32]. This practice likely aids the geographic spread of HPAI viruses and is a risky activity for poultry to human HPAI transmission [9].

Although not highlighted by this HACCP analysis, the review of existing literature also identified the care of poultry as a risk factor for HPAI H5N1 viral transmission from poultry to humans [22].

Introducing the preventative measures highlighted by this HACCP evaluation should reduce the occurrence of HPAI outbreaks. The parallel findings of our rapid HACCP assessment with the existing literature cited, provides strong evidence for the potential that HACCP analyses may have as a framework for helping local personnel to formulate a rapid response to an emerging health threat. Indeed, because the involvement of local personnel is a critical aspect of the HACCP process, we would argue that the process not only identifies key critical control points and suggests control strategies but provides those local personnel with the knowledge, understanding and ownership to more reliably implement any identified control measures.

### Scope for further application of the HACCP framework

The HACCP framework enables the identification of risks within a system and the design of control methods. It does not contain the scope for monitoring or ensuring compliance of the control points identified; such control should be applied via other means. Given that EIDs are appearing with increasing frequency, often in countries where they place additional strain on already over-burdened public health and healthcare systems, being able to rapidly identify and design strategies for control has valuable application in responding to emerging health threats such as the Middle East Respiratory Syndrome (MERS) virus which first appeared in Saudi Arabia in late 2012 [33] or the rapidly spreading outbreak of a novel avian influenza A H7H9 in China since March 2013 [34]. Conducting detailed, timely and comprehensive field investigations into HPAI H5N1 outbreaks is hampered by the majority of cases occurring in developing countries [35]. Advantages to such a framework are that it requires minimal resources and can be implemented by local health officials and international expertise, if required, can be provided remotely. It also complements recently developed diagnostic statistical models for known pathogens [36]. Subsequent detailed and time-consuming experimental analyses can then be conducted if required. Whereas in-depth epidemiological studies can take weeks or months to produce results and recommendations the HACCP framework may provide a means of producing a response within days of an outbreak occurring.

## References

[1] Karesh WB, Cook RA, Bennett EL, Newcomb J (2005) Wildlife trade and global disease emergence. Emerg Infect Dis 11: 1000-1002. PubMed: 16022772. 16022772 10.3201/eid1107.050194 PMC3371803

[2] Binder S, Levitt AM, Sacks JJ, Hughes JM (1999) Emerging Infectious Diseases: Public Health Issues for the 21^st^ Century. Science 284: 1311-1313. doi:10.1126/science.284.5418.1311. PubMed: 10334978. 10334978 10.1126/science.284.5418.1311

[3] Daszak P, Cunningham AA, Hyatt AD (2000) Emerging Infectious Diseases of Wildlife – Threats to Biodiversity and Human Health. Science 287: 443-449. doi:10.1126/science.287.5452.443. PubMed: 10642539. 10642539 10.1126/science.287.5452.443

[4] Chomel BB, Belotto A, Meslin F-X (2007) Wildlife, exotic pets and emerging zoonoses. Emerg Infect Dis 13: 6-11. doi:10.3201/eid1301.060480. PubMed: 17370509. 17370509 10.3201/eid1301.060480 PMC2725831

[5] Swift L, Hunter P, Lees A, Bell D (2007) Wildlife Trade and the Emergence of Infectious Diseases. Ecohealth 4: 25-30. doi:10.1007/s10393-006-0076-y.

[6] Jones KE, Patel NG, Levy MA, Storeygard A, Balk D, Gittleman JL et al. (2008) Global trends in emerging infectious diseases. Nature 451: 990-993. doi:10.1038/nature06536. PubMed: 18288193. 18288193 10.1038/nature06536 PMC5960580

[7] Weiss RA (2001) The Leeuwenhoek Lecture 2001 Animal origins of human infectious diseases, London School of Hygiene and Tropical Medicine. Phil Trans Roy Soc Lond. B. 356: 957-977

[8] Bell DJ, Roberton S, Hunter P (2004) Animals origins of SARS coronavirus: possible links with the international wildlife trade in small carnivores. Philos Trans R Soc Lond B. 359: 1107-1114. doi:10.1098/rstb.2004.1492. 15306396 10.1098/rstb.2004.1492 PMC1693393

[9] OIE, World Organisation for Animal Health Update on Highly Pathogenic Avian Influenza in animals 2013. Available. http://www.oie.int/animal-health-in-the-world/update-on-avian-influenza/2013/. Accessed: 20 April 2013

[10] Roberton SI, Bell DJ, Smith GJD, Nicholls JM, Chan KH et al. (2006) Avian Influenza H5N1 in Viverrids: implications for wildlife health and conservation. Proc R Soc Lond B. 273: 1729–1732. doi:10.1098/rspb.2006.3549. PubMed: 16790404. 10.1098/rspb.2006.3549 PMC1634780 16790404

[11] World Health Organization (WHO) (2013) Cumulative Number of Confirmed Human Cases of Avian Influenza A/(H5N1). Available: http://www.who.int/influenza/human_animal_interface/H5N1_cumulative_table_archives/en/index.html. Accessed: 20 April 2013.

[12] Thorson A, Petzold M, Chuc NTK, Ekdahl K (2006) Is Exposure to Sick or Dead Poultry Associated With Flulike Illness?: A Population-Based Study From a Rural Area in Vietnam With Outbreaks of Highly Pathogenic Avian Influenza. Arch Intern Med 166: 119–123. doi:10.1001/archinte.166.1.119. PubMed: 16401820. 16401820 10.1001/archinte.166.1.119

[13] Eagles D, Siregar ES, Dung DH, Weaver J, Wong F et al. (2009) H5N1 highly pathogenic avian influenza in Southeast Asia. Rev Sci Tech Off Int Epiz 28: 341–348. PubMed: 19618637. 10.20506/rst.28.1.1864 19618637

[14] Webster RG, Peiris M, Chen H, Guan Y (2006) H5N1 Outbreaks and Enzootic Influenza. Emerg Infect Dis 12: 3–8. doi:10.3201/eid1201.051024. PubMed: 16494709. 16494709 10.3201/eid1201.051024 PMC3291402

[15] Van Kerkhove MD, Mumford E, Mounts AW, Bresee J, Ly S et al. (2011) Highly Pathogenic Avian Influenza (H5N1): Pathways of Exposure at the Animal-Human Interface, a Systematic Review. PLOS ONE 6(1): e14582. doi:10.1371/journal.pone.0014582. PubMed: 21283678. 21283678 10.1371/journal.pone.0014582 PMC3025925

[16] Kung NY, Guan Y, Perkins NR, Bissett L, Ellis T et al. (2003) The impact of a monthly rest day on avian influenza virus isolation rates in retail live poultry markets in Hong Kong. Avian Dis 47: 1037–1041. doi:10.1637/0005-2086-47.s3.1037. PubMed: 14575106. 14575106 10.1637/0005-2086-47.s3.1037

[17] MacLehose L (2003) Hazard-Analysis-Critical-Control-Point-Methodologie. In: Krämer A Reintjes R Infektionsepidemiologie. Berlin, Heidelberg: Springer Verlag pp. 119-123.

[18] Edmunds KL (2011) Avian influenza, the wild bird trade and local livelihoods: an interdisciplinary and mixed methods approach. Unpublished PhD thesis, University of East Anglia, UK.

[19] Savill NJ, St Rose SG, Keeling MJ, Woodhouse MEJ (2006) Silent spread of H5N1 in vaccinated poultry. Nature 442: 757. doi:10.1038/442757a. PubMed: 16915278. 16915278 10.1038/442757a

[20] Tian G, Zhang S, Li Y, Bu Z, Liu P et al. (2005) Protective efficacy in chickens, geese and ducks of an H5N1-inactivated vaccine developed by reverse genetics. Virology 341: 153–162. doi:10.1016/j.virol.2005.07.011. PubMed: 16084554. 16084554 10.1016/j.virol.2005.07.011

[21] de Jong MD, Cam BV, Qui PT, Hien VM, Thanh TT et al. (2005) Fatal Avian Influenza A (H5N1) in a Child Presenting with Diarrhoea Followed by a Coma. N Engl J Med 352: 686-691. doi:10.1056/NEJMoa044307. PubMed: 15716562. 15716562 10.1056/NEJMoa044307

[22] Vong S, Ly S, Van Kerkhove MD, Achenbach J, Holl D et al. (2009) Risk Factors Associated with Subclinical Human Infection with Avian Influenza A (H5N1) Virus – Cambodia, 2006. J Infect Dis 199: 1744-1752. doi:10.1086/599208. PubMed: 19416078. 19416078 10.1086/599208

[23] Kandun IN, Samaan G, Harun S, Purba WH, Sariwati E et al. (2009) Chicken Faeces Garden Fertilizer: Possible Source of Human Avian Influenza H5N1 Infection. Zoonoses Public Health 57: 285-290. doi:10.1111/j.1863-2378.2009.01246.x. PubMed: 19912615. 19912615 10.1111/j.1863-2378.2009.01246.x

[24] Bridges CB, Lim W, Hu-Primmer J, Sims L, Fukuda K et al. (2002) Risk of influenza A (H5N1) infection among poultry workers, Hong Kong, 1997-1998. J Infect Dis 185: 1005–1010. doi:10.1086/340044. PubMed: 11930308. 11930308 10.1086/340044

[25] Mounts AW, Kwong H, Izurieta HS, Ho Y, Au T et al. (1999) Case-control study of risk factors for avian influenza A (H5N1) disease, Hong Kong 1997. J Infect Dis 180: 505–508. doi:10.1086/314903. PubMed: 10395870. 10395870 10.1086/314903

[26] World Health Organization (WHO) (2007) Review of the latest available evidence on potential transmission of avian influenza (H5N1) through water and sewage and ways to reduce the risks to human health. Available: http://www.who.int/water_sanitation_health/emerging/h5n1background.pdf. Accessed: 28 November 2012.

[27] Areechokchai D, Jiraphongsa C, Laosiritaworn Y, Hanshaoworakul W, O’Reilly M (2006) Investigation of Avian Influenza (H5N1) outbreak in humans--- Thailand, 2004. Centre for Disease Control, Morbidity and Mortality. pp. 3-6 Weekly Report 55 (Suppl 1) 16645574

[28] Beigel JH, Farrar J, Han AM, Hayden FG, Hyer R et al. (2005) Avian influenza A (H5N1) infection in humans. N Engl J Med 353: 1374–1385. doi:10.1056/NEJMra052211. PubMed: 16192482. 16192482 10.1056/NEJMra052211

[29] Dinh PN, Long HT, Tien NTK, Hien NT, Mai LTQ et al. (2006) Risk factors for human infection with avian influenza A H5N1, Vietnam, 2004. Emerg Infect Dis: 12: 1841–1847. doi:10.3201/eid1212.060829. PubMed: 17326934. 17326934 10.3201/eid1212.060829 PMC3291373

[30] Grenier M, Muller-Graf C, Hiller P, Schrader C, Gervelmeyer A et al. (2007) Expert opinion based modelling of the risk of human infections with H5N1 through the consumption of poultry meat in Germany. Berl Munch Tierarztl Wochenschr Heft 2/4: 98-107. 17416131

[31] Edmunds KL (2011) Avian Influenza, the Wild Bird Trade and Local Livelihoods: an Interdisciplinary and Mixed-Methods Approach. Unpublished PhD thesis, University of East Anglia, Norwich, UK.

[32] Liao QY, Lam WWT, Dang VT, Jiang CQ, Udomprasertgul V et al. (2009) What causes H5N1 avian influenza? Lay Perceptions H 5N1 aetiology in South East and East Asia. J Public Health 31: 573–581. 10.1093/pubmed/fdp043 19423546

[33] Promed (2012) PRO/AH/EDR> Novel coronavirus - Saudi Arabia (18): WHO, new cases, cluster 23/11/2012. Archive Number: 20121123.1421664. .

[34] Promed (2013) PRO/AH/EDR > Avian influenza, human (51): H7N9 update, 18/4/2013. Archive number: 20130418. 1655610

[35] Van Kerkhove MD, Ly S, Holl D, Guitian J, Mangtani P et al. (2008) Frequency and patterns of contact with domestic poultry and potential risk of H5N1 transmission to humans living in rural Cambodia. Influenza Other Respir Viruses 2: 155-163. doi:10.1111/j.1750-2659.2008.00052.x. PubMed: 19453420. 19453420 10.1111/j.1750-2659.2008.00052.x PMC4941898

[36] Bogich TL, Funk S, Malcolm TR, Chhun N, Epstein JH et al. (2013) Using network theory to identify the causes of disease outbreaks of unknown origin. J R Soc Interface 10(82) 20130127. doi:10.1098/rsif.2013.0127. PubMed: 23389893. 10.1098/rsif.2012.0904 PMC3627096 23389893

